# Establishment of an optimized guinea pig model of cisplatin-induced ototoxicity

**DOI:** 10.3389/fvets.2023.1112857

**Published:** 2023-04-13

**Authors:** Navid Ahmadi, Nodir Saidov, Julia Clara Gausterer, Anne-Margarethe Kramer, Clemens Honeder, Christoph Arnoldner

**Affiliations:** ^1^Department of Otorhinolaryngology, Head and Neck Surgery, Medical University of Vienna, Vienna, Austria; ^2^Division of Pharmaceutical Technology and Biopharmaceutics, Department of Pharmaceutical Sciences, University of Vienna, Vienna, Austria; ^3^Department of Biomedical Research, Medical University of Vienna, Vienna, Austria

**Keywords:** guinea pig model, protocol, cisplatin, ototoxicity, otoprotection

## Abstract

**Background:**

Cisplatin is among the most effective antineoplastic agents and has revolutionized the treatment of many cancer diseases. However, one of its serious side effects is a progressive and irreversible hearing loss, occurring in both adults and children. For the development of otoprotective therapies that prevent this side effect, cisplatin-induced hearing loss animal models are indispensable. Due to the high toxicity of cisplatin, the establishment of such animal models is a difficult and time-consuming task. Here we introduce the detailed protocol of a sophisticated guinea pig model with a sufficient and permanent hearing loss induced by cisplatin. This manuscript is intended to provide guidance in the development of future cisplatin guinea pig models which may reduce the mortality rate of the animals and help to gain more reproducible results.

**Methods:**

Pigmented and unpigmented guineapigs were treated with an intravenous single application of 8 mg/kg cisplatin under general anesthesia. An extensive and long-term intensive care protocol consisting of scheduled application of fluids, antiemetics, analgesics, glucose and supportive feeding among others, was used to ensure wellbeing of the animals. Hearing tests were performed prior to and 5 days after cisplatin application. Animals were then euthanized.

**Results:**

The ABR audiometry 5 days after cisplatin application revealed a hearing threshold ranging from 70 dB to 90 dB in the frequencies from 1 kHz to 32 kHz respectively.

All animals presented a good health condition despite the treatment with cisplatin.

**Discussion:**

The introduced care protocol in this manuscript is intended to serve as a guidance for the establishment of a stable guinea pig model for short- and long-term investigation regarding the inner ear and its protection in the frame work of cisplatin-induced damage.

## 1. Introduction

Cisplatin is among the most effective and frequently used antineoplastic agents. It is administered as chemotherapy for the treatment of various malignancies of the head and neck, lung, ovary, cervix, endometrium, pancreas, breast, and esophagus as well as melanomas, lymphomas, and metastatic osteosarcomas, among others (1). Its high cytotoxicity relies on covalent binding to the purine bases adenine and guanine, leading to intra-strand and inter-strand crosslinks with subsequent strand breaks. Its use has dramatically increased the survival and cure rate in both adults and children. However, it also involves numerous serious side effects. Progressive and irreversible hearing loss is among the most common long-term side effects, occurring in adults but particularly in children, who are more susceptible (2, 3). The consequences of hearing loss during childhood include impairment of speech and language acquisition, psychosocial and cognitive development, and educational and vocational achievement (2). More than 50% of patients with cancer suffer from permanent hearing loss following cisplatin chemotherapy (4). Although cisplatin is eliminated from most organs within days to weeks, it is retained for months to years after treatment in the vascular tissue of the cochlea and stria vascularis (5). This and the subsequent strial impairment may explain the susceptibility of the cochlea to cisplatin (5). Many immune suppressive, anti-oxidative, anti-apoptotic, and neurotrophic agents have been investigated during the last several decades for their potential to protect hearing during cisplatin chemotherapy (3, 6). To date, no methods or agents have been identified that completely protect cochlear structures and hearing from cisplatin-induced damage, in animal models or in the clinic. This fact emphasizes the importance of further experimental studies with well-established animal models in order to advance in the protection of the inner ear during platin based chemotherapy without counteracting its antineoplastic effects. However, the establishment of the respective animal model is a difficult undertaking due to the high toxicity of cisplatin. In many studies investigating cisplatin in guinea pigs, the health condition of the animals during the experiments, their mortality rate and the care protocol used to maintain an acceptable health condition in animals are not reported (7–13). In only few publications some of this information is contained. For example, Berglin et al. have reported their care protocol consisting of temgesic twice a day for analgesia and Ringer-Acetat solution for rehydration in a guinea pig model with 8 mg/kg intravenous single shot cisplatin application (14). Aslier et al. have reported that the animal wellbeing was maintained throughout the study in a guinea pig model with 14 mg/kg intraperitoneal single shot cisplatin application. The care protocol used in this study consisted saline twice daily for rehydration (15).

According to the experiences in our laboratory, the establishment of a cisplatin animal model is accompanied by various obstacles. First, there is a fine line between ototoxicity and lethality. Thus, a dose needs to be chosen which is high enough to induce a sufficient hearing loss on the one side and which can be tolerated by the animals for the whole duration of the study period on the other side. Due to its high toxicity, the application of cisplatin can be accompanied by a reduction of the general health condition, nausea, pain, kidney damage, reduced food intake, weight loss and premature death of the animals. Furthermore, the administration route itself may have an effect on the intensity of the side effects and complications. The adverse side effects may enforce a dose reduction during the study which in turn may lead to an insufficient manifestation of hearing loss in the animals. In order to keep the animals in a good condition throughout the study despite of the high- dose cisplatin application, special care of the animals needs to be taken. Fernandez et al. published a detailed protocol for a mouse model of cisplatin induced hearing loss (16). However, guinea pigs offer various advantages compared to mice. For example, the middle ear of guinea pigs is larger and their cochleae are more easily accessible, making them more suitable for experiments designed for investigation of locally applied drugs among others (17, 18). The audible frequency range of guinea pigs is similar to human which may facilitate the translation of the gained knowledge into clinical research (19). Furthermore, guinea pigs have been shown to be more susceptible to cisplatin induced hearing loss than mice (20). To the best of our knowledge, a detailed protocol of a guinea pig model with cisplatin induced hearing loss has not been described in the literature before.

Here we introduce a cisplatin guinea pig model with an optimized care protocol which allows to keep the animals in a desirable health condition for short- and long-term experimental studies in which cisplatin needs to be used. This protocol was developed in the framework of the establishment of a cisplatin induced hearing loss guinea pig model for the investigation of the otoprotective effects of an intratympanically applied agent. The aim of this manuscript is to provide a guidance on the development of cisplatin animal models which may reduce the mortality rate of the animals during the experiments and help to gain more reliable results.

## 2. Materials and equipment

The materials and equipment required are listed in Table 1.

**Table 1:** List of materials required.

**Reagents**	**Company**
Cisplatin	Ebewe Pharma Ges.m.b.H. Nfg. KG, Unterach, Austria
Cerenia^®^ (Maropitant)	Zoetis, Austria
Temgesic^®^ (Buprenorphine)	Indivior Europe Limited, Dublin, Ireland
Ketasol^®^ (Ketamine)	Livisto, Senden-Bösensell, Germany
Dormicum^®^ (Midazolam)	Roche, Vienna, Austria
Domitor^®^ (Medetomidin)	Orion Pharma, Vienna, Austria
Release^®^ (Pentobarbital)	WDT, Garbsen, Deutschland
NaCl 0.9%	Fresenius Kabi, Graz, Austria
Glucose 5%	B. Braun SE, Melsungen, Germany
Phosphate-buffered saline	Sigma Aldrich, Seelze, Germany
Buffered paraformaldehyde 4%	Sigma Aldrich, Seelze, Germany
**Equipment**	
Audiometric device	Intelligent Hearing Systems, Miami, USA
23 G blood collection needle	Vacuette^®^, Greiner Bio-One International, Kremsmuenster, Austria
26 G venous catheter	Neoflon. BD, Heidelberg, Germany
Syringe pump	B. Braun SE, Melsungen, Germany
Electric shaver	
Disinfectant	
Tourniquet	
Plaster	

## 3. Methods

This study was approved by the animal welfare committee of the Medical University of Vienna and the Austrian Federal Ministry for Science, Research and Economy (BMWFW-66.009/0397-WF/V/3b/2018).

Female pigmented guinea pigs (Core Facility, Medical University of Vienna, Himberg, Austria) and Dunkin-Hartley female albino guinea pigs (Charles River Laboratories, Sulzfeld, Germany) were included in the study as described below. The animals were given an adaptation time of at least 2 weeks after their arrival to our laboratory prior to the begin of the experiments. All experiments and measurements except for weighing were performed under general anesthesia. Prior to cisplatin application, auditory brainstem response (ABR) measurements were performed to exclude a pre-existing hearing disorder in all animals. On the day of cisplatin application, 50 μl of a thermoreversible poloxamer 407 hydrogel were applied intratympanically to the area of the round window niche using a YOU-1 micromanipulator (Narishige, Tokyo, Japan) and a Hamilton syringe (Hamilton, Bonaduz, Switzerland) with a blunt 29G needle. Another ABR measurement was then performed to keep record of a potential conductive hearing loss due to the gel application. Subsequently cisplatin was applied, while the animal still being under general anesthesia. In case of a persistent reduced condition animals were excluded from the study and euthanized. Prior to the euthanasia a final ABR was performed.

The development of the guinea pig model is summarized in chapter 5.1.

### 3.1. Final and validated cisplatin hearing loss guinea pig model

A venous catheter was placed in the saphenous vein. It can be found dorsal to the tarsal joint. Animals were restraint gently while leaving one of the legs accessible. The hairs covering the tarsal area were shaved with an electric shaver. The tarsal area was then disinfected after cleaning the leg with warm water. The leg of the guinea pig was extended while retaining the blood in the veins using a tourniquet to allow the saphenous vein become more visible. A 26 G venous catheter (Neoflon. BD, Heidelberg, Germany) was inserted in the vein and fixed with a plaster. Cisplatin ((SP-4-2)-diamminedichloridoplatinum (II), Ebewe Pharma Ges.m.b.H. Nfg. KG, Unterach, Austria) in a dose of 8 mg/kg was then applied slowly at a continuous speed by a syringe pump over a period of 30 min. Subsequently, the venous catheter was removed and the animal placed in a cage under an infrared lamp for the wake-up phase. 5 days after the cisplatin application, a final ABR test was performed and animals were then euthanized.

#### 3.1.1. Care protocol

At the beginning of the cisplatin application, 1 mg/kg maropitant ((2S,3S)-N-(5-tert-Butyl-2-methoxybenzyl)-2-(diphenylmethyl)-1-azabicyclo[2.2.2]octan-3-amine), a neurokinin-1 receptor antagonist antiemetic, was injected subcutaneously in the neck of the animal and a mixture of 12 ml/kg NaCl (sodium chloride) and 6 ml/kg 5% glucose was applied in the middle and upper part of the back of the animal using a 23G blood collection needle (Vacuette^®^, Greiner Bio-One International, Kremsmuenster, Austria). When the animal was woken up, 0.05 mg/kg Buprenorphine (Temgesic^®^, ((2S)-2-[(5R,6R,7R,14S)-17-cyclopropylmethyl-4,5-epoxy-6,14-ethano-3-hydroxy-6-methoxymorphinan-7-yl]-3,3-dimethylbutan-2-ol, Indivior Europe Limited, Dublin, Ireland) was injected subcutaneously. Maropitant was applied once daily. Buprenorphin and the mixture of NaCl and glucose were applied 3 times a day.

Animals were weighted daily. The behavior of the animals in terms of signs of pain or reduced general condition such as apathic behavior, refusal of food, erected hide, contorted body position, tachypnea or bradypnea were monitored several times a day. According to their feeding behavior animals were supportively fed with a special feed for weakened and anorectic herbivore (Herbi Care Plus, Garbsen, Germany) up to three times a day.

#### 3.1.2. General anesthesia

A mixture of 10 mg/kg ketamine ((RS)-2-(2-Chlorophenyl)-2-(methylamino)cyclohexanone), 1 mg/kg midazolam (8-chloro-6-(2-fluorophenyl)-1-methyl-4H-imidazo[1,5-a][1,4]benzodiazepine) and 0.3 mg/kg medetomidine ((RS)-4-[1-(2,3-Dimethylphenyl)ethyl]-3H-imidazole) was applied subcutaneously to the fat pad in the neck of the animal. If needed, ¼ of the initial dose can be given 30 min after the first injection to maintain sufficient anesthesia. At the end of the measurements or the experiment, the anesthesia was partially antagonized by 1 mg/kg atipamezole and 0.1 mg/kg flumazenil.

#### 3.1.3. Auditory brainstem response measurement

ABR measurements were performed in the frequency range of 1 kHz−32 kHz as described in detail elsewhere (21). Alternatively, audiometric measurements may also be performed as described by Yildiz et al. (22). In cases where no brainstem responses were measurable, the hearing threshold was defined as 100 dB.

#### 3.1.4. Euthanasia of the animals

Under general anesthesia (as described in chapter 2.3.1) animals were transcardially perfused with 50 mg/kg pentobarbital (5-Ethyl-5-(1-methylbutyl)-2,4,6(1H,3H,5H)-pyrimidinetrione). If harvesting of cochleae is intended, we instead recommend a transcardial perfusion with phosphate-buffered saline followed by buffered 4% paraformaldehyde (PFA, Sigma Aldrich, Seelze, Germany) in deep general anesthesia (12 mg/kg ketamine, 1.2 mg/kg midazolam, 0.3 mg/kg medetomidine and 0.036 mg/kg fentanyl).

### 3.2. Statistical analyses

Data are presented as mean ± standard deviation of mean. Independent *T*-test was used for comparisons between base line and post cisplatin data. A two-sided *p* < 0.05 was considered as significant. Analyses were performed using IBM SPSS Statistics Version 28 (IBM Corp., Armonk, USA) and Microsoft Excel version 16.

## 4. Results

Thirty six female pigmented and albino guinea pigs were used to establish and validate the animal model. The final protocol was performed on 12 albino guinea pigs. Table 1 provides an overview of the characteristics of the animals used.

### 4.1. Establishment of the cisplatin hearing loss guinea pig model

**Figure 1:**
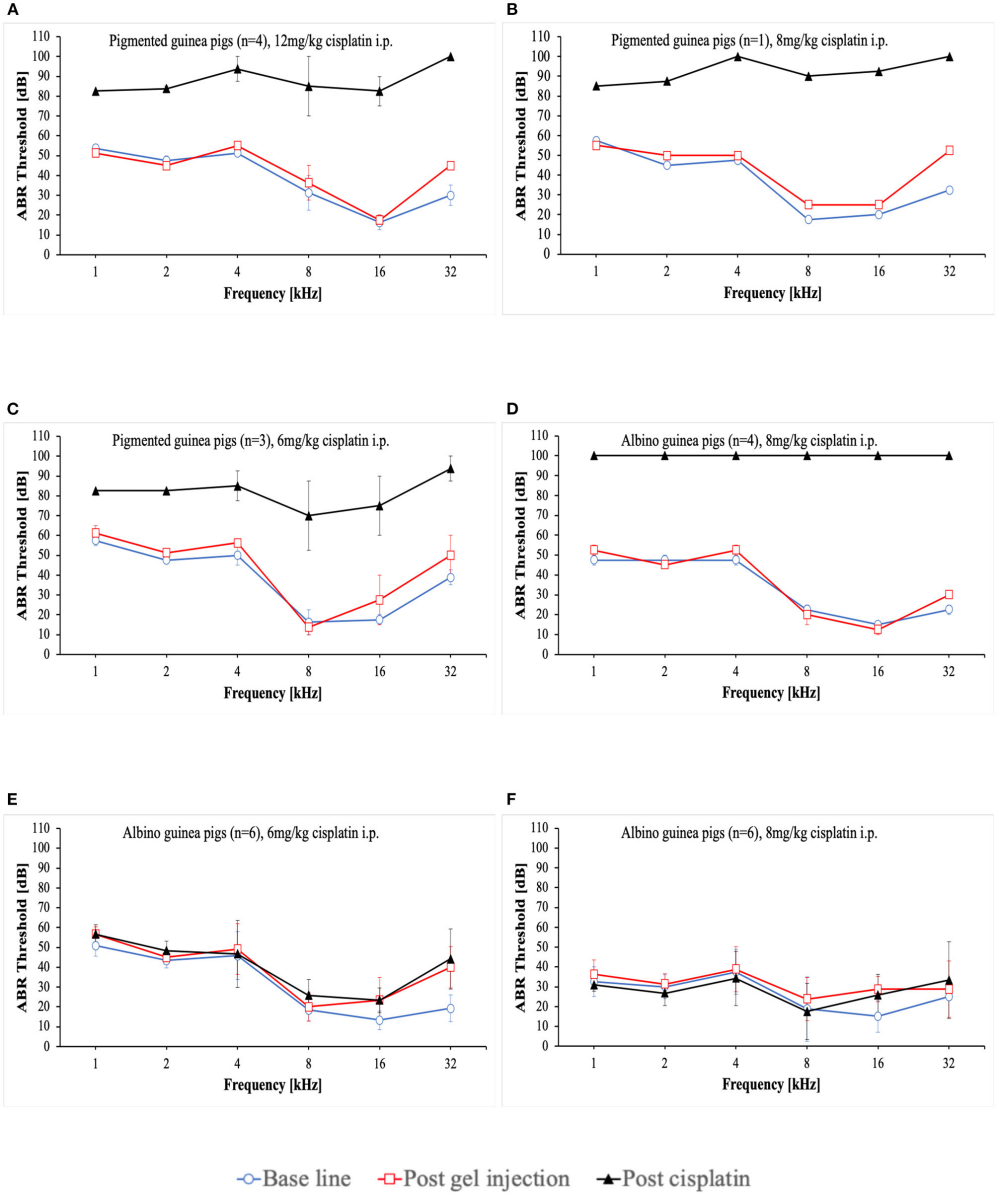
Mean ABR thresholds at base line, after intratympanic hydrogel injection and at day 3 **(A–C)** or day 5 **(D–F)** after cisplatin application. Error bars are presented as standard deviation.

At the start of the project, four pigmented guinea pigs at age of 10 to 12 weeks weighting ~700 g received 12 mg/kg cisplatin intraperitoneally (i.p.) (Table 2). Analgesic therapy with 0.05 mg/kg buprenorphine (Temgesic^®^) was performed in two animals during the first 2 days after cisplatin application. No regular fluid substitution was performed. All animals developed a bad general condition and died between day 1 and 6 after cisplatin. As shown in Figure 1A, a mean severe to profound hearing loss was developed in all frequencies. In two animals no ABR could be performed due to the bad condition of the animals. Two animals developed a hematuria. In one animal an autopsy was performed which revealed signs of peritonitis.

**Table 2:** Characteristics of the animals.

**Race**	**Number**	**Dose [mg/kg]**	**Application route**	**Weight [g] ±SD**	**Weight loss [%]**	**Bad condition [%]**	**Survival [days] ±SD**
Pigmented	4	12	i.p.	703 ± 82	12 ± 12	100 (*n* = 4/4)	3 ± 1
Pigmented	1	8	i.p.	840	8.5	100 (*n* = 1/1)	3
Pigmented	3	6	i.p.	774 ± 148	12^*^	100 (*n* = 3/3)	5 ± 3
Albino	4	8	i.p.	674 ± 22	10 ± 9	100 (*n* = 4/4)	3 ± 1
Albino	6	6	i.p.	622 ± 41	16 ± 4	16 (*n* = 1/6)	5 ± 0
Albino	6	8	i.p.	373 ± 26	2 ± 7	50 (*n* = 3/6)	4 ± 0
Albino	12	8	i.v.	406 ± 30	8.7 ± 4	0	5 ± 0

Weight refers to the time point immediately before the cisplatin application. SD, standard deviation. ^*^, the weight of only one animal could be measured prior to death.

That followed, one pigmented guinea pig received a reduced cisplatin dose of 8 mg/kg i.p. (Table 2). Analgesic therapy with 0.05 mg/kg buprenorphine (Temgesic^®^) and fluid substitution with ringer's lactate solution was performed during the first 3 days after cisplatin application. This animal developed also a persistent bad condition and was euthanized on day 3. Figure 1B shows the respective ABR result.

In the subsequent three animals, a further dose reduction of cisplatin to 6 mg/kg i.p. was established. Analgesic therapy with 0.05 mg/kg buprenorphine (Temgesic^®^) was performed on some days according to the condition of the animals and pain signs. Fluid substitution was performed irregularly either with ringer's lactate solution, glucose solution or NaCl. A supportive feeding was started in the last two animals in this group. One animal died on day 10, one on day 5 and the other one on day 2. In the last-mentioned animal, no final ABR could be performed. The ABR results are presented in Figure 1C.

In further consequence, while the care protocol was continuously optimized, a change to younger albino guinea pigs aged a~9 weeks took place (Table 2). 8 mg/kg cisplatin was applied i.p. to 4 animals. In this group a regular analgesic therapy (0.05 mg/kg buprenorphine (Temgesic^®^) three times a day) as a fix part of the care protocol was established. Fluid substitution was performed with either ringer's lactate solution or NaCl. Supportive feeding was performed in some animals according to the observed condition of the animals. The animals still showed a bad general condition. One animal developed a diarrhea. One animal needed to be euthanized on day 4. The rest died between days 2 and 6. Two animals died before a final ABR could be performed. In the other two animals no hearing threshold could be detected on day 3 (Figure 1D). An autopsy of one animal again showed signs of peritonitis.

In further consequence again a dose reduction to 6 mg/kg cisplatin was established in six guinea pigs (Table 2). Ringer's lactate solution was given three times a day during the first 2 days after cisplatin application. A regular supportive feeding regimen three times a day during the first 3 days after cisplatin application was established from now on. As a new part of the care protocol an antiemetic therapy (1 mg/kg maropitant once a day) during the first 3 days after cisplatin application was included. Only one of the animals presented itself with a reduced condition and a bloody urine. All animals survived until day 5 and were then euthanized according to the protocol. In these animals no hearing threshold shift was detected 5 days after cisplatin making the investigation of the effects of potential otoprotective substances impossible (Figure 1E).

Therefore, for the next six animals a dose increase to 8 mg/kg cisplatin i.p. was established while performing the advanced care protocol (Table 2). The decision was made to include younger animals at age of ~5 to 6 weeks. In these animals a mixture of NaCl and Glucose solution was applied three times a day until day 5. Supportive feeding was performed in all of them three times a day until day 5 according to their needs. Three of six animals showed a reduced health condition despite of doing better than the former animals. Two of them died at day 3. One of them was euthanized on day 4. The remaining three animals showed a good condition and were euthanized on day 5 according to the protocol. However, in these animals still no relevant hearing threshold shift could be detected 5 days after cisplatin application (Figure 1F).

**Figure 2:**
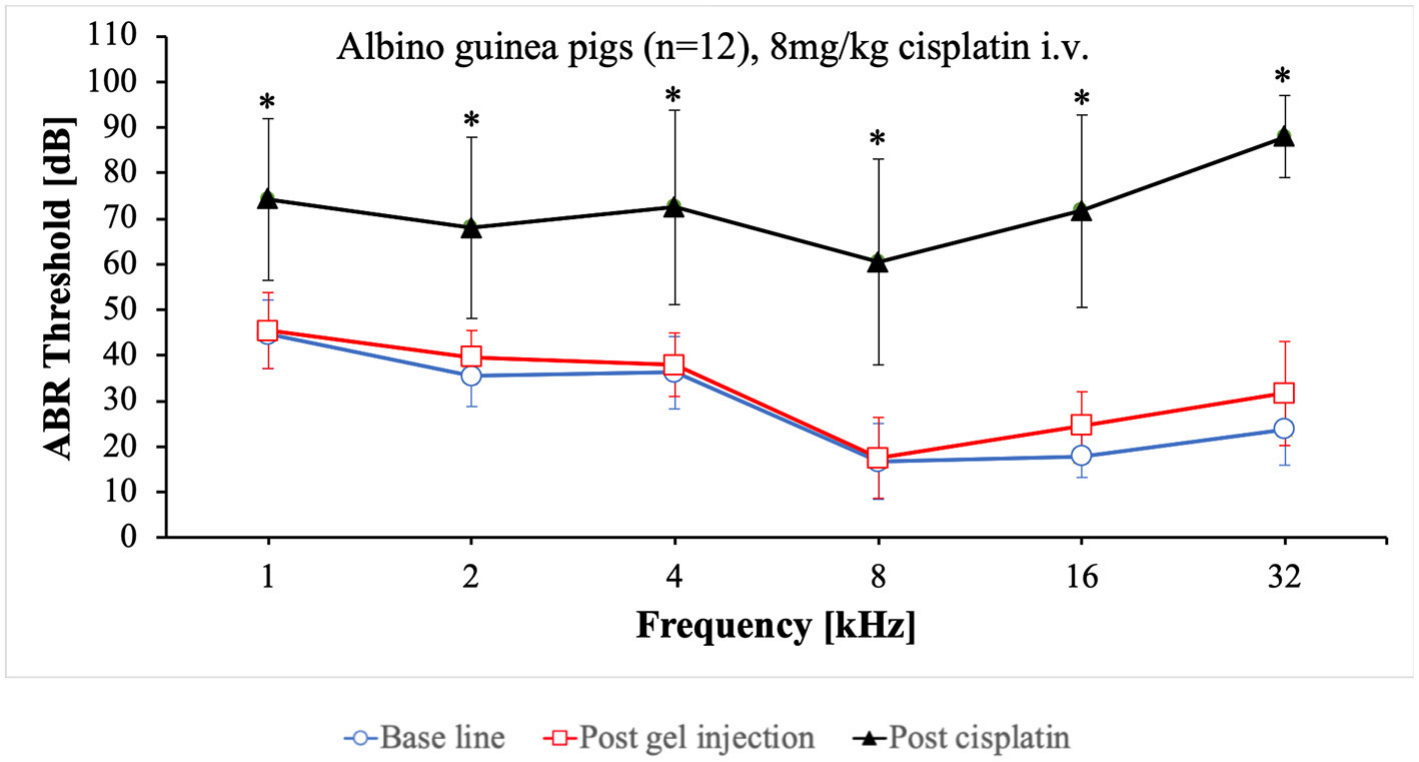
Mean ABR thresholds at base line, after intratympanic hydrogel injection and at day 5 after cisplatin application. Error bars are presented as standard deviation. ^*^Significant difference between base line and post cisplatin (*p* ≤ 0.001).

With the gradually matured care protocol, the application route was changed from intraperitoneal to intravenous while the dose of 8 mg/kg cisplatin remained unchanged (Table 2). Cisplatin was slowly applied at a continuous speed by a syringe pump over a period of 30 min. The final care protocol implemented is described in chapter 4.1.1. All of the animals (*n* = 12) showed a good condition and were euthanized on day 5. The ABR audiometry revealed a sufficient hearing loss ranging from 70 dB to 90 dB (Figure 2). The threshold shift was significant in all frequencies tested. The individual threshold shifts of the animals treated with the final care protocol are presented in the Supplementary Figure 1.

## 5. Discussion

In the last decades, many studies using cisplatin animal models have been published (3, 23). In the field of otology, the investigation of the pathophysiology of the ototoxicity of platinum-based agents and its prevention while maintaining their antineoplastic effects has been a main focus of research. To the best of our knowledge, in studies employing cisplatin models, the general health condition of the animals during the experiments, their mortality rate, their exclusion rate—for example due to an undesirable severe or insufficient hearing loss -, the arduousness that the authors may have experienced and the protocol for maintaining the animals in an at least stable condition have not been reported. However, this information is of great value as it may enable other researchers to establish a similar animal model according to their own research questions in a shorter amount of time and with a lower number of animals used for this purpose.

In this manuscript, we describe a detailed protocol to establish a stable and reproduceable cisplatin guinea pig model for various research questions. This protocol was developed in close collaboration with the veterinary doctors in our animal research laboratory. The challenge in dealing with animals receiving cisplatin relies on the high toxicity of this chemotherapeutic agent. Cisplatin may lead to acute kidney injury (24). Consistent with this fact we observed that some of our animals developed a hematuria during the experiments. In order to reduce the nephrotoxicity of cisplatin we established a concomitant regular fluid substitution beginning simultaneously with the slow cisplatin application. We initially used isotonic NaCl and Ringer's solution. However, with the combination of NaCl and glucose the best results in terms of the condition of the animals could be reached.

Nausea is another common adverse side effect of chemotherapeutic agents which we also observed in our animals (25). This side effect is of utmost importance as it leads to a food rejection of animals which in turn can lead to a rapid deterioration of their general condition. We applied the antiemetic active ingredient Maropitant already at the beginning of the cisplatin application to counteract a possible food deprivation due to nausea after the animals woke up.

Cisplatin may lead to pain (26). The choice of the right analgesic and its frequent application as it is suggested here should not be underestimated. We used Buprenorphine in our study due to its high analgetic efficacy, good tolerability and especially due to the fact that there are no otic or nephrotoxic effects known (27). Non-steroidal anti-inflammatory drugs (NSAID) are also effective analgesics and well-tolerated in animals (28). However, in the field of otologic research they may not be suitable as they may lead to a drug induced hearing loss (29). On the other hand, some authors have suggested that this drug class may also exhibit a protective effect on the inner ear (30, 31). Therefore, NSAID may have an impact on the results of studies carried out in this field. The nephrotoxic effect of NSAIDs is another reason for their inappropriateness in studies with cisplatin as they may represent an additional burden for the kidneys (32).

According to our observations, additional factors playing an important role in the management of animals receiving cisplatin are the age and weight of the animals. One could imagine that older animals with a higher bodyweight may be more resilient to the toxic effects of cisplatin. However, the opposite was the case in our experiments. The younger and more lightweight our animals were, the better was their condition following cisplatin application. Due to the fact that younger animals were at the same time lighter than the older animals, the impact of each weight and age on its own remains speculative.

Intraperitoneal application of cisplatin has been used in many hearing loss animal models (15, 33–36). It has been shown that this application route leads to a high local concentration of cisplatin with a relatively low systemic absorption and toxicity (37, 38). The low toxicity is for sure preferable, although the low systemic absorption may result in an insufficiently low hearing loss (Figures 1E, F). Intraperitoneal application of cisplatin is for sure eligible in the therapy of intraperitoneal malignancies (39), but its appropriateness in the development of animal hearing loss models for the investigation of otoprotective agents remains questionable. We have shown that after improving the care protocol, no relevant hearing threshold shift was achieved with 8 mg/kg cisplatin i.p. whereas the same dosage led to a sufficient hearing loss when applied intravenously (i.v.) (Figure 1F). It shouldn't remain unmentioned that the intraperitoneal application has several drawbacks. It may lead to a gut perforation with subsequent peritonitis and sepsis. Peritonitis may also occur without gut perforation as it was observed in our study. This leads to abdominal pain and plays a considerable negative role in the general condition of the animals resulting in food refusal and rapid weight loss. The change of the application route from intraperitoneal to intravenous represented an important advancement in the development of our cisplatin animal model. Together with this step we included a daily subcutaneous application of an antiemetic and an extension of the period of the analgetic therapy from 3 to 5 days. These protocol changes enabled us to maintain all our animals in a good health condition over the whole study period.

Additionally, we observed that the health condition and hydration of the animals is associated with the ABR-results. Animals receiving 8 mg/kg and 6 mg/kg cisplatin i.p. at the beginning of the study developed a severe hearing loss, while animals receiving the same dosages later during the course of the study didn't show any hearing loss. Most of the latter animals already exhibited a relatively good condition due to the advanced care protocol while the former ones developed a worsening of health condition. Especially the former animals receiving 8 mg/kg cisplatin i.p. revealed a severe bad condition. In these animals no hearing threshold could be determined at all (Figure 1D) which is in accordance with the observed association between the health condition and ABR-results. Schmutzhard et al. and Fischer et al. have shown that sepsis leads to a significant hearing impairment in mice which may further emphasize the relation between general health status of the animals and their hearing ability (40, 41). A similar phenom is also seen in critically ill patients (42). However, the exact pathophysiology in this context needs to be investigated in further studies.

The function of melanocytes and their level in different guineapig races are considerable factors. In addition to organs such as skin and eyes, these cells are also found in in the stria vascularis and modiolus of the inner ear (43). Melanocytes are involved in various intracellular metabolic pathways and are important for the development of a normal hearing (43, 44). In the inner ear melanocytes are suggested to be protective against reactive oxygen species as well as noise and drug induced hearing loss (45–47). For examples albino guinea pigs have been shown to be more susceptible to noise induced hearing loss when compared to pigmented guinea pigs (48). However, in this study a differing susceptibility between pigmented and albino guineapigs was not observed.

Finally, we want to emphasize the importance of supportive feeding. Despite of a well-wrought care protocol, cisplatin remains a highly cytotoxic agent. Some animals may develop a loss of appetite and subsequent food refusal. Such a behavior should be recognized quickly. In dependance of the animal's own food intake, a gentle supportive manual feeding up to three times a day can be performed. This procedure is well accepted by the animals and counteracts an uncontrolled weight loss and reduction of the general health condition.

## 6. Conclusion

The establishment of a cisplatin animal model is time consuming and difficult due to the high toxicity of this agent. Here we introduced for the first time a detailed, sophisticated and validated protocol for a cisplatin guinea pig model that can be used for research questions considering the inner ear and its protection. We also have described the procedure of its development and the setbacks we have experienced on this way. This information may be valuable for future experiments in this area. This protocol was developed on basis of the pathophysiology of cisplatin toxicity and the experiences of the authors. The establishment of a cisplatin animal model begins with the right choice of the weight of the animals. Furthermore, it includes a special care schedule for the guinea pigs consisting of regular investigation of their general health condition, sufficient fluid administration, analgesic and antiemetic therapy as well as supportive manual feeding according to their individual needs.

## Data availability statement

The raw data supporting the conclusions of this article will be made available by the authors, without undue reservation.

## Ethics statement

The animal study was reviewed and approved by Animal Welfare Committee of the Medical University of Vienna and the Austrian Federal Ministry for Science, Research and Economy (BMWFW-66.009/0397-WF/V/3b/2018).

## Author contributions

NA performed the animal experiments, developed the care protocol, and analyzed the date and prepared the manuscript. NS contributed equally to the animal experiments and the implementation of the care protocol and involved in the evaluation of the data. A-MK made the veterinary consultations and played a substantial role in the development of the care protocol. JG, CH, and CA supported the project with their knowledge and experience. All authors contributed to the article and approved the submitted version.

## Supplementary material

Supplementary Figure 1 Individual ABR threshold shifts of guinea pigs treated with the final care protocol on day 5 after cisplatin application.
