# Supplementary figures and images for: Establishment of an optimized guinea pig model of cisplatin-induced ototoxicity
Source: Front Vet Sci. 2023 Apr 13;10:1112857. doi: 10.3389/fvets.2023.1112857 (PMC10133486; doi:10.3389/fvets.2023.1112857)

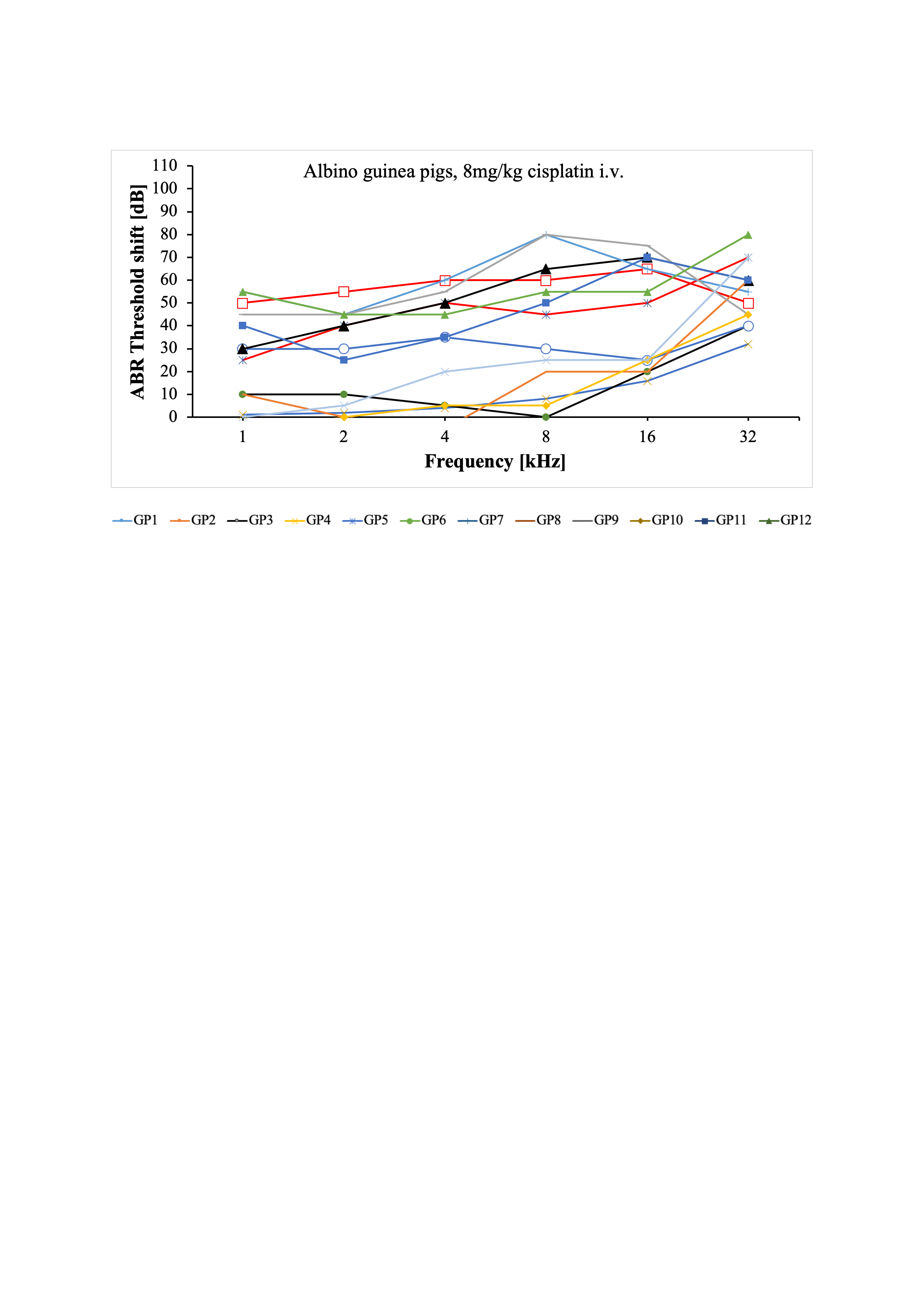

Supplement: Supplementary Figure 1 — Individual ABR threshold shifts of guinea pigs treated with the final care protocol on day 5 after cisplatin application. [file Image_1.TIFF]
